# Protein-directed nucleotide selection in the DRT3 defense system: mechanism, biological context, and constraints on programmability

**DOI:** 10.3389/fmicb.2026.1866040

**Published:** 2026-06-16

**Authors:** Sarfaraz K. Niazi

**Affiliations:** Pharmaceutical Sciences, College of Pharmaceutical Sciences, Washington State University, Spokane, WA, United States

**Keywords:** bacterial anti-phage immunity, central dogma of molecular biology, defense-associated reverse transcriptase, dinucleotide-repeat DNA, DRT3 defense system, protein-directed nucleotide selection, ribonucleoprotein complex, template-independent DNA synthesis

## Abstract

The DRT3 bacterial defense system reveals a previously undocumented mode of specificity in DNA synthesis, wherein a protein active site, rather than a complementary nucleic acid strand, dictates nucleotide incorporation. This mechanism necessitates a precise spatial configuration. DRT3 does not encode a protein-to-DNA sequence transfer; rather, it produces a singular repetitive product, an alternating poly (GT/AC) double-stranded DNA, whose sequence is constrained by the geometry of the Drt3b active site. Structural and biochemical analyses of the reconstituted Escherichia coli system reveal a ribonucleoprotein complex comprising two reverse-transcriptase-like proteins (Drt3a and Drt3b) and a non-coding RNA scaffold, assembled into a D3-symmetric 6:6:6 architecture at 2.2–2.6 Å resolution. Drt3a synthesizes the poly (GT) strand utilizing a conserved RNA motif as a Watson–Crick template, whereas Drt3b generates the complementary poly (AC) strand independently of a nucleic acid template, consistent with the absence of templating nucleic-acid density in its active site. This review contextualizes the discovery of DRT3 within the broader evolution of DNA synthesis biochemistry and defense-associated reverse transcriptases and elucidates the mechanistic foundation of protein-directed nucleotide selection. Three primary constraints are emphasized: First, the system demonstrates structural control of sequence specificity without programmability; there is no evidence for the engineering of alternative motifs. Second, no therapeutic application based on DRT3 has been validated in mammalian systems or *in vivo* models. Third, any translational extension would require resolving fundamental issues related to enzyme persistence, intracellular DNA accumulation, off-target innate immune activation, and genotoxicity. Consequently, the DRT3 system signifies an expansion of the mechanistic landscape of DNA synthesis chemistry, with substantial conceptual implications, although its biotechnological potential remains dependent on future engineering and validation efforts.

## 1 Introduction

**Figure 1:**
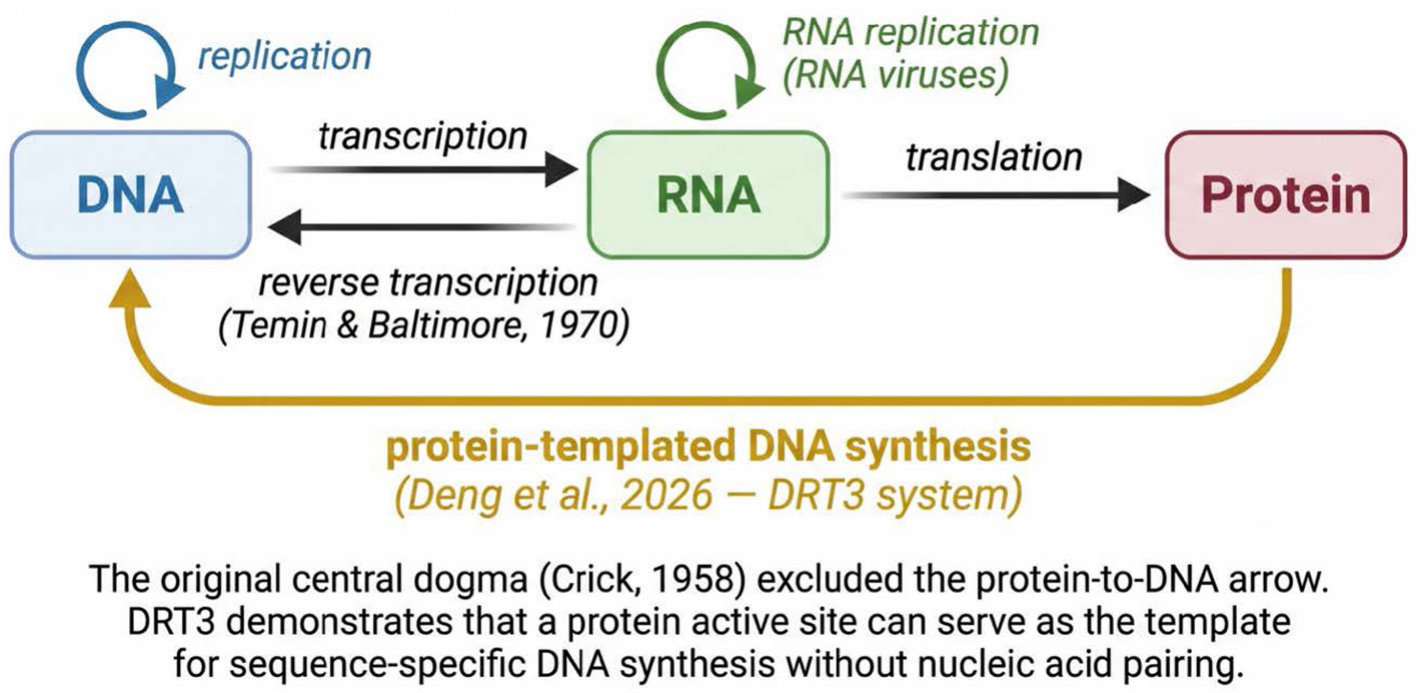
The central dogma of molecular biology and its subsequent revisions. Crick (1958, 1970) articulated the transfer of genetic information within biological systems as the permissible and impermissible flow of sequence information between DNA, RNA, and proteins. The discovery of reverse transcription, independently identified by Temin and Baltimore in 1970, confirmed that RNA-to-DNA information transfer occurs naturally. The DRT3 system does not introduce a new category of forbidden transfer into this framework; rather, it exemplifies a structural mode of specificity: a protein active site can enforce sequence specificity in DNA synthesis independently of a complementary nucleic acid template. The amino acid sequence of the protein is not transcribed into the DNA sequence, and the natural product involved in this process is a single, repetitive species, specifically an alternating poly(GT/AC) DNA. Accordingly, the figure should be interpreted as broadening the understanding of permissible transfers without contravening the fundamental logic of the central dogma. Created in BioRender. Niazi, S. (2026).

Since its molecular discovery in the mid-20th century, DNA synthesis has traditionally been regarded as a process involving a nucleic acid template (Watson and Crick, 1953; Kornberg, 1957). The principle of base-pairing, whereby a nucleotide is incorporated into a growing strand through complementarity with an opposing nucleotide, underpins fundamental biological processes such as replication, transcription, and reverse transcription. Notably, reverse transcription, which necessitated a revision of the central dogma following its identification within the genomes of RNA tumor viruses (Temin and Mizutani, 1970; Baltimore, 1970), still requires that a nucleic acid strand encode the sequence of the resulting product. The recent identification of the DRT3 system in Escherichia coli, published in Science in April 2026, has revealed a biochemical class in which sequence specificity in DNA synthesis is determined by the geometry of a protein active site rather than by an opposing nucleic acid strand. Figure 1 contextualizes this discovery within the ongoing revisions of the central dogma of molecular biology.

This finding must be articulated with caution, as it resides at a juncture where exaggeration is easily achievable. DRT3 is not a system in which the amino acid sequence of a protein is transcribed into a DNA sequence following a coding relationship. The relationship between the protein sequence and the DNA sequence is structural rather than encoding specific conserved side chains within the Drt3b active site, which occupy positions typically occupied by the opposing strand, thereby influencing nucleotide incorporation toward adenine in one cycle and cytosine in the subsequent cycle. The resulting product is a singular, fixed, repetitive species that alternates between GT and AC dinucleotides on opposite strands, extending on a kilobase scale. Currently, there is no evidence to suggest that the system can be engineered to generate arbitrary sequences. Furthermore, the natural product itself appears to lack any discernible coding capacity (Deng et al., 2026).

The discovery of DRT3 represents the culmination of a research trajectory that commenced with the systematic computational identification of bacterial defense systems (Doron et al., 2018; Gao et al., 2020). In 2020, Gao and colleagues at the Broad Institute reported that bacteria encode a significantly greater number of anti-phage immune systems than previously anticipated, with a considerable proportion of these systems containing reverse transcriptases of unknown function (Gao et al., 2020). Subsequent investigations expanded the catalog of these defense-associated reverse transcriptases (DRTs) and uncovered mechanistic features that diverge from the classical reverse-transcription paradigm (Mestre et al., 2022). The DRT2 system was identified as assembling repetitive cDNA copies of a non-coding RNA into an extrachromosomal gene encoding a toxic protein, in a process that mirrors and extends the logic of rolling-circle replication (Wilkinson et al., 2024; Tang et al., 2024). The DRT4 system was demonstrated to be a single-gene, template-independent polymerase, whose toxic single-stranded DNA product is stabilized in the presence of a phage-encoded protein, ORF55 (Wang et al., 2026). The DRT9 system was discovered to synthesize long poly (A)-rich complementary DNA in response to phage-induced alterations in deoxynucleotide triphosphate pools, and to form a hexameric ribonucleoprotein assembly (Han et al., 2025; Song et al., 2025; Tang et al., 2025). Each new DRT subtype has progressively broadened the conceptual understanding of the potential functions of reverse transcriptases.

The DRT3 system, as characterized by Deng and colleagues at Stanford, advances the trajectory to a further and qualitatively distinct stage (Deng et al., 2026). The system synthesizes a double-stranded DNA composed of alternating GT and AC dinucleotides. One strand is synthesized by Drt3a, utilizing the non-coding RNA component as a conventional template in a manner mechanistically reminiscent of telomerase (Greider and Blackburn, 1985; Blackburn and Collins, 2011). The other strand is produced by Drt3b in the absence of any nucleic acid template. Cryo-electron microscopy density maps do not reveal a strand of RNA or DNA occupying the position typically held by an opposing template within the Drt3b active site; instead, this position is occupied by amino acid side chains that constrain the next nucleotide to be incorporated. This mode of templating, which the authors designate as protein-templated DNA synthesis, lacks precedent in the existing literature on nucleic acid polymerases (Deng et al., 2026).

The purpose of this review is threefold. First, to position the discovery of DRT3 within the historical and conceptual context of DNA synthesis biochemistry, including the gradual relaxation of the central dogma over more than half a century, thereby enabling an objective assessment of the implications of this finding rather than a sensationalized depiction (Crick, 1958, 1970; Cobb, 2017). Second, to provide a detailed description of the DRT3 system as documented, supplemented by the broader landscape of DRT, retron, and reverse-transcriptase-based defense mechanisms (Gao et al., 2020; Mestre et al., 2022). Third, to outline the translational potential, acknowledging that the natural product is a fixed, repetitive species, that programmability has not been verified, and that no preclinical proof-of-concept for DRT3-based therapeutics has been published. The material is organized into four figures and three tables. Throughout, the objective is to remain faithful to the published data, while clearly delineating where speculation begins.

## 2 Historical context: from watson and crick to protein-directed nucleotide selection

### 2.1 The original central dogma and its formal commitments

Crick's original statement of the central dogma in 1958 was a forecast that turned out to be largely correct: it predicted that the residue-by-residue sequence of nucleic-acid bases would be translatable into the residue-by-residue sequence of amino acids in proteins, but that the reverse, namely transfer of information from a defined protein sequence to a defined nucleic-acid sequence, would not occur in living systems (Crick, 1958; Cobb, 2017). The 1970 reformulation, written in response to the discovery of reverse transcription, partitioned information transfers into general, special, and forbidden classes (Crick, 1970). General transfers, including DNA-to-DNA replication, DNA-to-RNA transcription, and RNA-to-protein translation, occurred routinely in cells. Special transfers included RNA-to-DNA reverse transcription, RNA-dependent RNA replication, and direct DNA-to-protein translation; these were demonstrably possible but restricted to contexts. Forbidden transfers were those for which Crick saw no biochemical mechanism and whose occurrence would, he suggested, require a substantial revision of molecular biology. The principal forbidden transfer was protein-to-nucleic-acid, by which he meant the transfer of the amino-acid sequence of a defined protein into the nucleotide sequence of a defined nucleic acid. The discovery of DRT3 does not violate this prohibition. The amino acid sequence of Drt3b is not transcribed into the DNA product; rather, the DNA product's sequence is not derived from a residue-by-residue readout of the protein. Instead, the three-dimensional conformation of Drt3b forms an active-site cavity whose geometry influences nucleotide incorporation in an alternating pattern. The DNA sequence is thus determined by the architecture of the protein, as the cleavage specificity of a restriction enzyme is dictated by its structure, although it is not directly encoded by the sequence itself (Deng et al., 2026). This distinction is fundamental and is reiterated multiple times throughout this review, as it is where common misunderstandings often arise. The precise statement is that DRT3 exemplifies protein-directed nucleotide selection, rather than protein-templated information transfer in the sense articulated by Crick (Figure 1).

### 2.2 Reverse transcription and the first revision of the dogma

The discovery of reverse transcriptase in 1970 by Temin and Baltimore marked the first official revision of the central dogma (Temin and Mizutani, 1970; Baltimore, 1970; Coffin and Fan, 2016). This discovery emerged from research on RNA tumor viruses, particularly Rous sarcoma virus, whose biology presented a paradox: the virus was composed of RNA, yet its propagation appeared to require integration into the host genome, which consisted of DNA. The resolution was that the virion contained an enzyme capable of synthesizing a DNA copy from the RNA template. The biochemical and clinical implications of this finding were significant, ultimately facilitating the development of zidovudine, lamivudine, tenofovir, and the broader class of nucleoside reverse transcriptase inhibitors, which remain central to the management of human immunodeficiency virus infection (De Clercq, 2004). Crick himself revised the central dogma in 1970, recognizing RNA-to-DNA transfer as a permitted special case, and the wider scientific community readily accepted this modification, partly because the mechanism preserved the fundamental logic of base-pair complementarity (Crick, 1970).

The finding regarding DRT3 is conceptually distinct from reverse transcription in a manner worthy of emphasis. Reverse transcription adheres to the principle that a sequence of bases on one strand guides the sequence of bases on the complementary strand through Watson-Crick base pairing. Conversely, the DRT3 mechanism departs from this principle for one of the two strands. Specifically, the Drt3a-mediated synthesis of the poly (GT) strand is conventional, RNA-templated, and reliant on Watson-Crick complementarity, whereas the Drt3b-mediated synthesis of the poly (AC) strand does not adhere to this principle. In the case of Drt3b, the active-site cavity, shaped by conserved residues identified by Deng et al. (2026), occupies the position typically occupied by an opposing template, aligning with discrimination among the four potential nucleotides at each step. Structural data corroborate this interpretation, as no nucleic acid density is observed at the position normally occupied by a templating base (Deng et al., 2026).

### 2.3 RNA catalysis and the broadening of biochemical possibility

A subsequent noteworthy precedent is the discovery of catalytic RNA by Cech and Altman in the early 1980s, which demonstrated that nucleic acids alone could catalyze reactions previously attributed solely to protein enzymes (Kruger et al., 1982; Guerrier-Takada et al., 1983). The identification of ribozymes did not directly contradict the central dogma; rather, it transformed molecular biologists' understanding of the relationship between sequence, structure, and function. Moreover, it provided conceptual support for the RNA world hypothesis, suggesting that early life forms utilized RNA both as genetic material and as a primary catalyst prior to the development of the dual-molecule system characteristic of modern biology (Gilbert, 1986; Joyce, 2002). The discovery of DRT3 aligns well with these conceptual developments, further reducing the presumed distinction between proteins as catalysts and nucleic acids as informational molecules. In summary, each incremental advancement, from reverse transcription and ribozymes to prion-mediated propagation of structural states and presently, protein-guided nucleotide selection, has enriched the understanding of biological chemistry without fundamentally challenging the core principles of base-paired replication.

## 3 The DRT family of bacterial anti-phage systems

### 3.1 Bacterial immunity beyond CRISPR

The discovery of CRISPR-Cas systems and their adaptation as gene-editing tools has garnered considerable public attention in the field of molecular biology for over a decade; however, bacteria possess a significantly broader array of anti-phage defense mechanisms beyond CRISPR alone (Doron et al., 2018; Tesson et al., 2022; Georjon and Bernheim, 2023). Comprehensive computational and experimental investigations conducted over recent years have identified more than 100 distinct defense systems, many of which are encoded within defense islands in bacterial genomes and utilize an impressive diversity of biochemical strategies (Doron et al., 2018; Vassallo et al., 2022). These include restriction-modification systems, abortive infection systems, retrons, CBASS systems utilizing cyclic nucleotide signaling, and defense-associated reverse transcriptases (DRTs), which are the primary focus of this review. Each of these systems represents a distinct solution to the same evolutionary challenge: namely, how to detect and respond to phage infection before the phage can complete its lytic cycle (Hampton et al., 2020).

The DRT systems were initially recognized as a distinct category in 2020, when Gao and colleagues demonstrated that several previously uncharacterized bacterial reverse transcriptases endowed resistance to phages when heterologously expressed in Escherichia coli (Gao et al., 2020). The comprehensive survey identified nine DRT subtypes, labeled DRT1-DRT9, differentiated by their domain architecture, gene neighborhood, and the presence or absence of a non-coding RNA component (Mestre et al., 2022). Some DRTs function as single-gene systems, with the reverse transcriptase protein alone being sufficient for defense; others necessitate an associated non-coding RNA, and a few rely on additional protein partners. Across all variants, the catalytic activity of the reverse transcriptase domain remains essential, although the chemical nature and biological roles of the DNA products differ (Gonzalez-Delgado et al., 2021; Mestre et al., 2022).

### 3.2 Mechanistic diversity within the DRT family

The mechanistic characterization of individual DRT systems has lagged their initial functional identification; however, in the past 2 years, a series of high-resolution studies has begun to elucidate the details. The DRT2 system from ^*^Klebsiella pneumoniae^*^, characterized concurrently by the Sternberg laboratory at Columbia University and the Zhang laboratory at the Broad Institute, was shown to assemble a long, repetitive cDNA molecule via rolling-circle reverse transcription of a small non-coding RNA (Tang et al., 2024; Wilkinson et al., 2024). Phage infection triggers a transition from single-stranded to double-stranded synthesis, resulting in a lengthy DNA molecule whose adjacent repeats reconstitute a promoter and an open reading frame. The resulting transcript encodes a repetitive protein, designated Neo, which demonstrates high toxicity to the host cell and induces a state of dormancy, thereby aborting the phage replication cycle (Wilkinson et al., 2024). The mechanism through which a non-coding RNA is transcribed into a coding DNA gene via reverse transcription was unprecedented at the time of its discovery.

**Figure 2:**
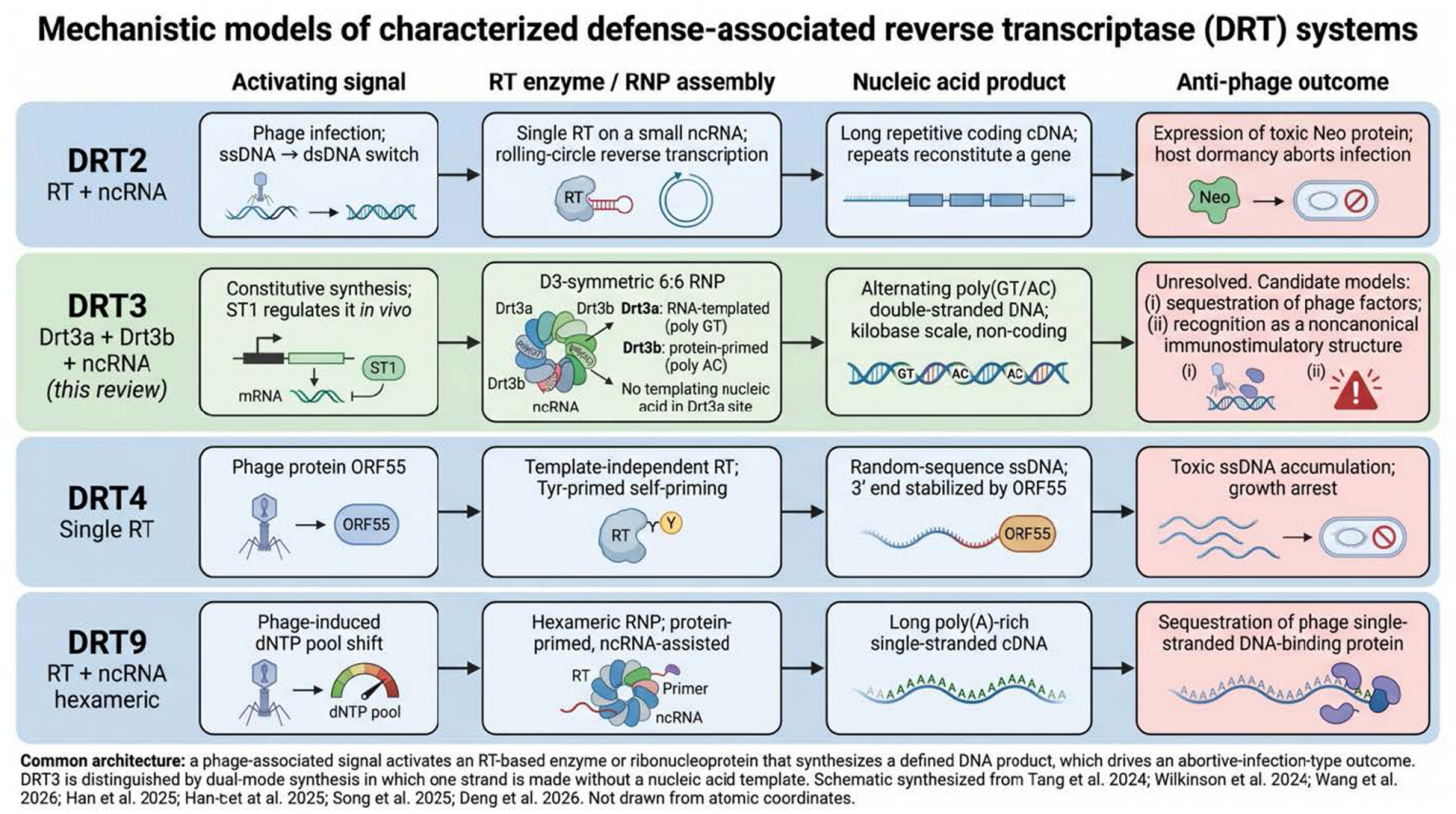
A comparative diagram of the characterized DRT-family anti-phage defense mechanisms. Each row depicts a system starting with its activating signal (on the left) and proceeding through the reverse transcriptase enzyme or ribonucleoprotein assembly, culminating in the nucleic acid product and the subsequent anti-phage response (on the right). DRT2 reverse-transcribes a non-coding RNA via a rolling-circle mechanism to generate a repetitive coding cDNA, whose translation leads to the synthesis of the toxic Neo protein and host dormancy. DRT3 (highlighted) assembles a D3-symmetric 6:6:6 ribonucleoprotein complex in which Drt3a synthesizes the poly(GT) strand on an RNA template, whereas Drt3b synthesizes the complementary poly(AC) strand without requiring a templating nucleic acid in its active site; this results in a kilobase-scale alternating poly(GT/AC) duplex, whose defensive role remains to be elucidated. DRT4 is a single, template-independent reverse transcriptase that accumulates toxic single-stranded DNA stabilized by the phage protein ORF55. DRT9 forms a hexameric ribonucleoprotein complex that synthesizes long poly(A)-rich single-stranded cDNA, which sequesters the phage single-stranded DNA-binding protein. The schematic provides a conceptual synthesis based on the cited primary literature and is not derived from atomic coordinates.

The DRT4 system, in contrast, is a single-gene system whose protein synthesizes a single-stranded DNA template independently (Wang et al., 2026). The protein contains a conserved tyrosine residue that serves as the priming site for DNA synthesis, in a self-priming mechanism reminiscent of the protein-primed replication used by certain bacteriophages such as phi29 (Salas, 1991). Phage infection triggers the production of a phage-encoded DNA-binding protein, ORF55, which protects the three-prime end of the DRT4 product from host exonucleases, thereby allowing the accumulation of toxic single-stranded DNA and arresting cell growth (Wang et al., 2026). The DRT9 system, independently characterized by two research groups in 2025, was demonstrated to form a hexameric ribonucleoprotein complex and to synthesize long poly (A)-rich single-stranded cDNA in response to phage-induced fluctuations in deoxynucleotide triphosphate concentrations (Han et al., 2025; Song et al., 2025; Tang et al., 2025). The poly (A) product is believed to sequester essential phage single-stranded DNA-binding proteins. The DRT6 system has been characterized at low resolution and appears to share architectural features with DRT4, with ORF55 also acting as its activator (Wang et al., 2025). The DRT7/UG10 systems have been described in a preprint reporting cryo-electron microscopy structures and polymerization activity; however, the mechanism remains under investigation at the time of writing (Figiel et al., 2026, preprint). To facilitate comparison across these diverse systems, Figure 2 presents a consolidated schematic model of the characterized DRT defense mechanisms, tracing each from the activating signal through enzyme assembly and DNA product to the resulting defensive outcome. Table 1 subsequently delineates the corresponding mechanistic characteristics and contrasts them with retrons and the well-established CRISPR-Cas systems. Collectively, the figure and table demonstrate that the DRT family exhibits heterogeneity, with no single DRT subtype serving as a representative of the others, and that DRT3 remains the most distinctive member to date.

**Table 1:** Mechanistic comparison of characterized defense-associated reverse transcriptase systems and adjacent bacterial defense modalities.

System	Components	DNA product	Templating mode	Defense mechanism	Reference
DRT2	RT + ncRNA	Repetitive coding cDNA, kilobase-scale	RNA-templated, rolling circle	Expression of toxic Neo protein, dormancy	Tang et al., 2024; Wilkinson et al., 2024
DRT3	Drt3a + Drt3b + ncRNA, 6:6:6 complex	Alternating poly(GT/AC) dsDNA, kilobase-scale	Mixed: RNA-templated (Drt3a) and protein-directed (Drt3b)	Unresolved; candidate models include sequestration of phage factors or recognition as a non-canonical structure (Deng et al., 2026)	Deng et al., 2026
DRT4	Single RT	Random-sequence ssDNA	Template-independent, protein-primed (Tyr)	Toxic ssDNA accumulation; activated by phage ORF55	Wang et al., 2026
DRT6	RT (DRT4 homolog)	Not yet fully characterized	Probably similar to DRT4	Activated by phage ORF55; mechanism unresolved	Wang et al., 2025
DRT7/UG10 [PREPRINT, provisional]	RT	Polymerase activity demonstrated	Under investigation	Anti-phage defense; structural basis reported	Figiel et al., 2026 (preprint)
DRT9	RT + ncRNA, hexameric	Long poly(A)-rich ssDNA	Protein-primed, ncRNA-assisted	Sequestration of phage SSB protein	Han et al., 2025; Song et al., 2025; Tang et al., 2025
Retrons (Ec86, Ec107, etc.)	RT + msr-msd ncRNA	Multicopy single-stranded DNA (msDNA), short	RNA-templated, branched RNA-DNA primer	Toxin/antitoxin, abortive infection	Millman et al., 2020; Bobonis et al., 2022
CRISPR-Cas *(contextual comparator; not a DRT system)*	Cas effector + crRNA	Not synthesized; cleaves DNA	RNA-guided sequence recognition	Cleavage of foreign DNA or RNA	Doudna and Charpentier, 2014

The table is restricted to systems for which biochemical characterization has been published. Several other DRT subtypes (DRT1, DRT5, DRT8) are known to confer phage resistance but remain unresolved at the mechanistic level. DRT7/UG10 information is from a preprint and should be treated as provisional. References cite the most informative primary report; for systems characterized by multiple groups in parallel, both reports are listed.

## 4 The DRT3 system: architecture, mechanism, and functional output

### 4.1 Discovery, cloning, and reconstitution

The DRT3 system was initially identified as a phage-resistance locus within the survey conducted by Gao et al. (2020). In this study, a heterologous expression strategy was employed to assess the activity of computationally predicted defense systems in ^*^Escherichia coli^*^. The system was characterized by the presence of two reverse-transcriptase-like proteins, a configuration that classified it within the UG3 plus UG8 grouping of the broader unknown-group reverse-transcriptase classification (Mestre et al., 2022). Additionally, a small non-coding RNA was discovered downstream of the protein-coding genes, and all three components were demonstrated to be essential for conferring phage protection (Gao et al., 2020). For several years, the molecular mechanisms underlying this system remained unresolved, partly because heterologously expressed DRT3 produced DNA species whose origins were difficult to reconcile with conventional reverse transcription chemistry (Mestre et al., 2022; Deng et al., 2026).

The Stanford group, led by Alex Gao with first authors Pujuan Deng, Hyunbin Lee, and Carlo Armijo, undertook a systematic biochemical and structural characterization of the DRT3 system from Escherichia coli (Deng et al., 2026). The plasmids encoding the system have been deposited in Addgene, enabling other laboratories to reproduce and extend the work. Heterologous expression in E. coli, followed by purification of the assembled complex and tagmentation sequencing of the DNA products, allowed the authors to identify the natural product as alternating poly (GT/AC) double-stranded DNA, present at a kilobase scale even in the absence of phage infection (Deng et al., 2026). The production of this product was constitutive rather than phage-induced, indicating that the catalytic activity of the complex is on by default and that phage detection acts downstream of DNA generation rather than as a trigger for it.

### 4.2 Cryo-electron microscopy reveals a 6:6:6 hexameric assembly

Cryo-electron microscopy of the purified DRT3 complex at an overall resolution of 2.6 angstroms revealed that the system assembles into a D3-symmetric ribonucleoprotein complex comprising six copies each of Drt3a, Drt3b, and the non-coding RNA (Deng et al., 2026). The structural organization features an outer ring of Drt3a subunits, encircling an inner ring of Drt3b subunits, with the non-coding RNA components positioned along the assembly's central axis. The synthesized double-stranded DNA was observable in the maps at the complex's periphery, consistent with a model suggesting that both strands are extruded during synthesis. The structural data were sufficiently robust to assign each component of the complex unambiguously. Significantly, the absence of any nucleic acid strand at the templating position of the Drt3b active site constitutes a notable finding, as it provides the sole structural evidence supporting the hypothesis of protein-mediated nucleotide selection.

**Figure 3:**
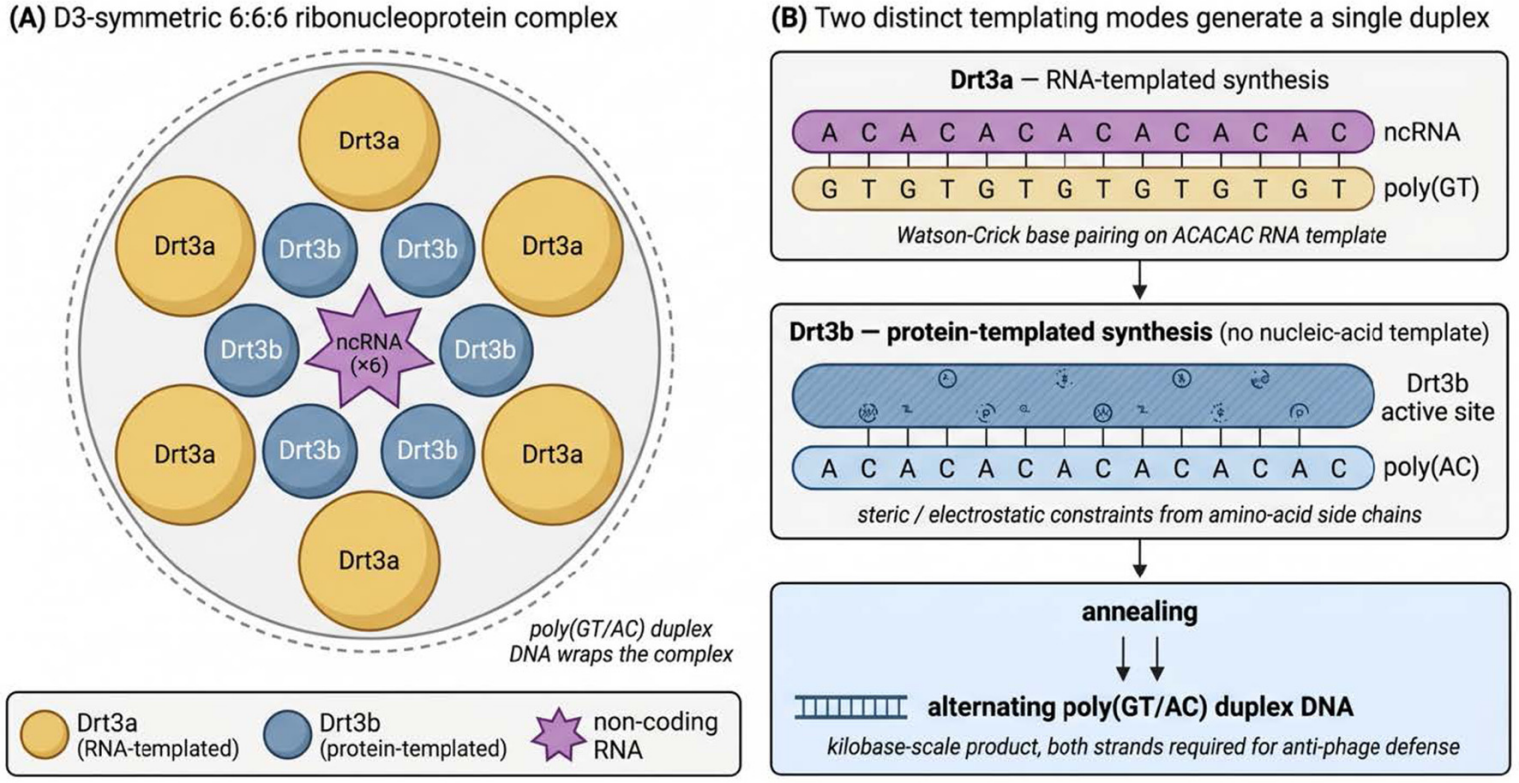
Schematic representation of the architecture and dual-mode catalysis of the DRT3 ribonucleoprotein complex, derived from the published cryo-electron microscopy structure. This illustration is not a reproduction of the structural data nor is it based on modified atomic coordinates; rather, it serves as a schematic depiction that emphasizes the key topological and mechanistic features. **(A)** displays a top view of the D3-symmetric 6:6:6 assembly, composed of six copies of the outer Drt3a subunit, six copies of the inner Drt3b subunit, and the central non-coding RNA scaffold. The synthesized poly(GT/AC) duplex DNA is depicted at the periphery, consistent with the published structural model. **(B)** demonstrates the two distinct templating modes: Drt3a utilizes a conserved ACACAC motif within the non-coding RNA as a Watson-Crick template to generate the poly(GT) strand, whereas Drt3b employs conserved active-site residues to regulate nucleotide incorporation and independently synthesize the complementary poly(AC) strand without a nucleic-acid template. The two strands anneal to form a kilobase-scale duplex, which is essential for anti-phage activity. This description is based on experimental data presented in Deng et al. (2026); interested readers are referred to that publication for detailed structural information.

Figure 3 illustrates the architecture of the DRT3 complex and the dual templating modes it employs. The illustration is a conceptual interpretation derived from the published cryo-electron microscopy structure, rather than a direct reproduction of the raw structural data. Panel A presents the D3-symmetric 6:6:6 configuration, which has no direct precedent among previously characterized reverse-transcriptase complexes. Although this hexameric organization is unusual, it shares certain features with the DRT family; for instance, DRT9 has been demonstrated to form a hexameric complex with its non-coding RNA, albeit with a different architecture (Han et al., 2025; Song et al., 2025). Panel B depicts the two distinct strand-synthesis activities. Drt3a functions as a conventional RNA-templated reverse transcriptase, reading the conserved ACACAC motif within the non-coding RNA and synthesizing the complementary poly(GT) strand via Watson-Crick base pairing. Conversely, Drt3b operates independently of a nucleic acid template, with conserved active-site residues occupying positions typically held by an opposing strand, thereby guiding nucleotide incorporation toward alternating adenine and cytosine through steric and electrostatic constraints (Deng et al., 2026).

### 4.3 The biochemical logic of protein-directed nucleotide selection

The biochemical mechanism by which the Drt3b active site enforces sequence specificity is conceptually distinct from any previously documented for nucleic acid polymerases. Conventional polymerases utilize the geometry of the templated strand, in conjunction with the geometry of the incoming nucleotide, to discriminate among the four possible bases at each position; the predominant chemistry involves Watson-Crick hydrogen bonding, supplemented by minor-groove contacts and shape selectivity (Kornberg and Baker, 1992; Kunkel and Bebenek, 2000). In Drt3b, discrimination is achieved by amino acid side chains that imitate, in chemical terms, the contacts made by a templating base. Conserved residues within the active site contribute to nucleotide discrimination, with mutational disruption of these residues abolishing the alternating incorporation pattern; a fully resolved residue-by-residue model explaining how adenine and cytosine are selected during alternating cycles has not yet been established (Deng et al., 2026). Consequently, the geometry of the protein, rather than that of a nucleic acid strand, determines the sequence alternation of the product. It is important to underscore the distinction highlighted throughout this review. The amino acid sequence of Drt3b is neither transcribed nor translated into a DNA product in a direct manner; the relationship between the protein and DNA sequences does not constitute a coding relationship comparable to the genetic code. Instead, the three-dimensional conformation of Drt3b creates an active-site cavity whose geometry exclusively permits a specific pattern of alternating dinucleotides. Mutations that modify the relevant residues abolish the alternation pattern, indicating that the specificity is encoded within side chains rather than in the overall geometry of the active site. Consequently, the DRT3 system exemplifies protein-directed nucleotide selection, a structural form of specificity that complements, rather than replaces, the base-pairing logic inherent in traditional polymerases. The remaining questions are whether other DRT subtypes utilize similar mechanisms and whether this principle can be generalized to enzymes outside this family.

### 4.4 The role of the non-coding RNA

The non-coding RNA component of DRT3 performs at least two essential functions. First, it serves as the template for Drt3a, with the conserved ACACAC motif providing the basis for poly(GT) synthesis via canonical reverse transcription. Second, it assumes a structural role; the six copies of this non-coding RNA are centrally arranged within the hexameric complex and appear vital for the integrity of the assembly (Deng et al., 2026). This dual functionality, serving both as a template and a scaffold, is analogous to the non-coding RNA components observed in telomerase and CRISPR-Cas systems, where the RNA element's role extends beyond simple information transfer (Blackburn and Collins, 2011; Jinek et al., 2012). In DRT3, the structural importance of the non-coding RNA is particularly significant, as it orchestrates spatial coordination between the two reverse transcriptase species, thereby ensuring that the synthesized strands anneal into a single duplex without disintegrating the assembly.

### 4.5 The defensive mechanism: open questions

The role of the DRT3 product in anti-phage defense has yet to be definitively determined. Two non-exclusive hypotheses have been proposed within the primary literature (Deng et al., 2026). The first is a sponging model, in which the kilobase-scale repetitive duplex sequesters phage-encoded factors that bind microsatellite-like sequences, thereby inhibiting the phage's access to its substrates. The second is a recognition model, proposing that the atypical repetitive duplex functions as an immunostimulatory signal, engaging bacterial pattern-recognition systems analogous, conceptually, to the cyclic GMP-AMP synthase pathway observed in vertebrates (Sun et al., 2013). Evidence from comparative studies of related DRT subtypes is suggestive but not conclusive: DRT9 sequesters phage single-stranded DNA-binding protein via a poly(dA) product (Han et al., 2025), and DRT2 induces dormancy by translating a toxic Neo protein from a reverse-transcribed gene (Tang et al., 2024; Wilkinson et al., 2024). The distinctive dinucleotide-repeat structure of the DRT3 product, which differs from that of other subtypes, may interact with sensors or binding partners that lack clear counterparts in other DRT systems. Consequently, direct experimental validation is now of utmost priority. The phage-induced regulator ST61 has been identified as a probable activator of DRT3 anti-phage activity *in vivo*; however, the relationship between the complex's constitutive DNA-generating activity and the regulated step remains to be elucidated (Deng et al., 2026).

## 5 Conceptual implications for molecular biology

### 5.1 What the DRT3 finding does and does not change

The discovery of DRT3 broadens the range of recognized chemistries involved in biological information transfer; however, it does not fundamentally challenge the central dogma. Crick (1970) explicitly addressed the question of whether a protein could dictate the sequence of a nucleic acid and concluded that no biochemical mechanism to such an effect had been established. The DRT3 system provides a previously undocumented or rarely observed mechanism of this kind, albeit in a limited form: the protein active site enforces a fixed alternating dinucleotide pattern rather than an arbitrary sequence dictated by the amino acid-by-amino acid order. The most precise articulation is that DRT3 demonstrates protein-directed nucleotide selection, in which the sequence of the synthesized DNA is determined by the geometry of a protein's active site rather than by an opposing nucleic acid template. This constitutes a genuine conceptual advancement; however, it is more constrained than some popular interpretations of this discovery have suggested. Clarifying this distinction is significant, as it delineates the system's capabilities and limitations as a foundational element for biotechnological applications.

### 5.2 DNA as a functional polymer rather than only a genetic carrier

An additional conceptual point reinforced by the DRT3 finding is that DNA is not solely a carrier of genetic information. The repetitive duplex generated by DRT3 possesses minimal informational content; it cannot encode a protein nor serve as a substrate for transcription or translation within the bacterial host. Its function is primarily structural or interactive, contingent upon which model of the defensive mechanism is validated (Deng et al., 2026). In this context, the DRT3 product aligns with an expanding list of biological DNA entities, whose functions are physical or signaling rather than informational. These include extrachromosomal circular DNAs and other DNA species recognized by innate immune sensors, such as cyclic GMP-AMP synthase, in which the molecular signal derives from the presence of DNA in an inappropriate cellular compartment rather than from the specific nucleotide sequence (Sun et al., 2013; Chen et al., 2016). This conceptualization of DNA also finds expression in DNA nanotechnology, in which DNA serves as a programmable building material for the construction of three-dimensional structures, hydrogels, and dynamic nanomachines (Seeman and Sleiman, 2018; Li et al., 2024; Ma et al., 2024).

### 5.3 A note on origin-of-life implications

The finding related to DRT3 has been cited in the origin-of-life discourse, as it demonstrates that protein structure can direct nucleic acid synthesis independently of an opposing template (Joyce, 2002; Müller et al., 2022). This association is briefly acknowledged, given that the DRT3 system is most convincingly interpreted as a relatively recent adaptation for anti-phage defense, rather than as a remnant of early biochemistry. Furthermore, this inquiry is ancillary to the primary focus on translation within this review.

## 6 Translational implications: a bounded outlook

To date, no DRT3-based therapeutic concept has been translated into preclinical practice in any disease model. As of this writing, no published study has demonstrated DRT3 expression or activity in mammalian cells, no engineered Drt3b variant producing a non-natural motif has been reported, and no *in vivo* biodistribution, pharmacokinetic, or efficacy data exist for any DRT3 component. The discussion below is therefore an outline of conceptual possibilities and engineering requirements rather than a development roadmap; the application spaces it surveys would each require prior demonstration of programmability, function in mammalian cells, and defined *in vivo* behavior before they could be considered candidate development programs.

### 6.1 The pharmacological logic of *in situ* DNA generation

The therapeutic utilization of the DRT3 mechanism would not involve the direct application of the natural enzyme. Instead, it would deliver an mRNA encoding an engineered DRT3 enzyme, encapsulated within a lipid nanoparticle, which, in turn, produces a specific DNA species *in situ*. This approach distinguishes itself from traditional oligonucleotide drugs, such as CpG immunostimulants, transcription factor decoys, and antisense oligonucleotides, which are chemically synthesized and delivered in their intact form (Krieg, 2002; De Stefano, 2011; Crooke et al., 2021). *In situ* generation facilitates enzymatic amplification, spatial restriction to the sites of enzyme expression, and a temporal linkage between expression and effect. However, these same properties introduce certain risks: amplification of an immunologically active DNA species is inherently difficult to dose-control, as the pharmacokinetics of the active species depend on enzyme persistence, mRNA stability, and DNA degradation, rather than on the administered dose. Accumulation of cytosolic DNA locally could activate the cyclic GMP-AMP synthase and stimulator of interferon genes pathway, whose uncontrolled activation has been associated with severe inflammatory phenotypes, including type I interferonopathies (Decout et al., 2021). Therefore, containment strategies must be addressed prior to exploiting the amplification advantage.

### 6.2 Hypothetical application spaces

**Figure 4:**
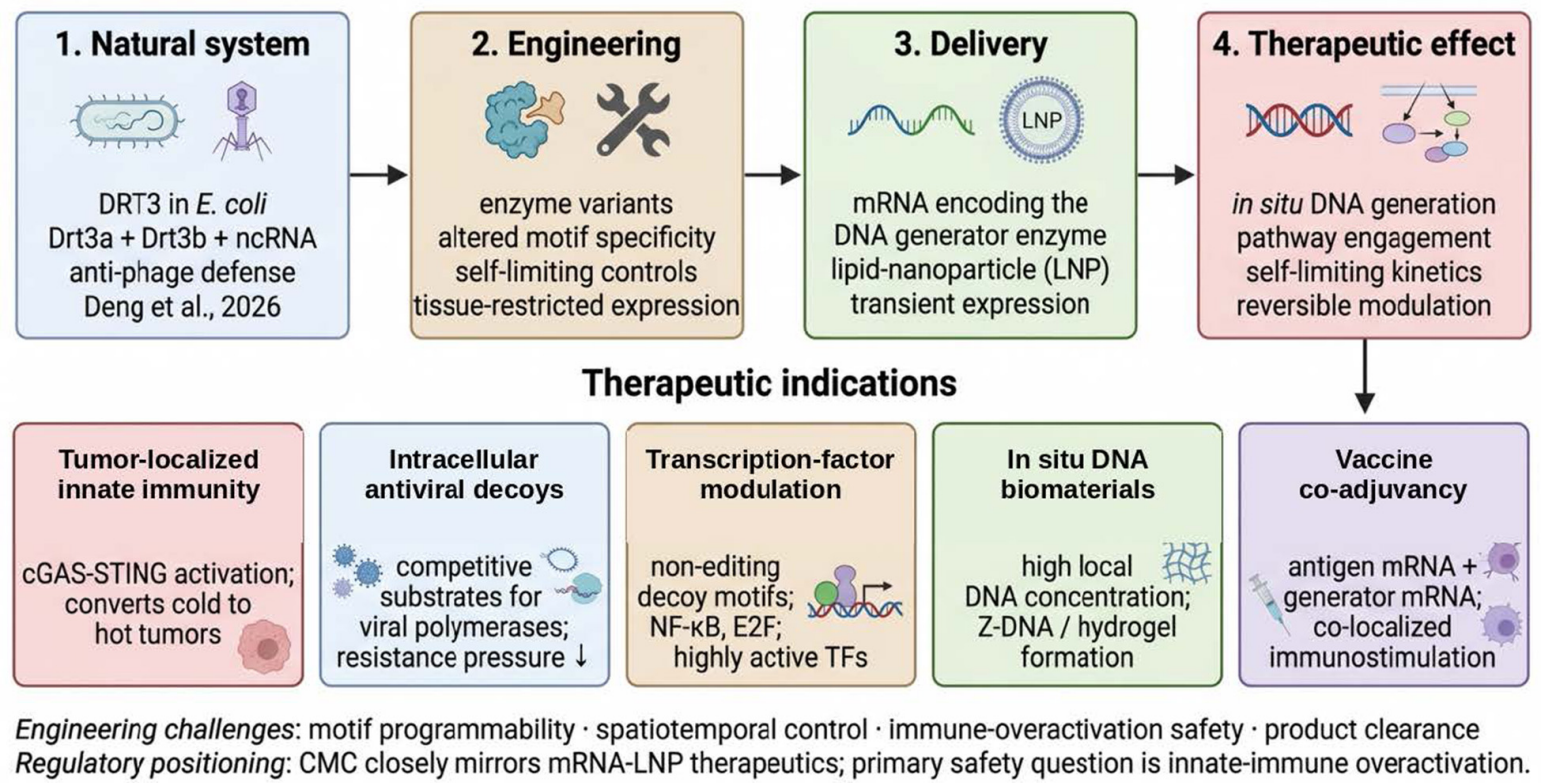
Hypothetical translational framework for the DRT3 platform. The upper row illustrates the four conceptual stages of development required for any therapeutic deployment. The lower row delineates five hypothetical application spaces proposed in early commentary. It is important to note that none of these applications has been experimentally demonstrated with the DRT3 system; thus, the figure should be regarded as an organizational scaffold for translational reasoning rather than as a depiction of validated programs. The lower annotations identify principal engineering challenges and highlight that, although the regulatory positioning shares surface features with mRNA-LNP biologics, it would require a distinct characterization package to address the produced DNA, biodistribution, persistence, and off-target innate immune activation.

Five application spaces have been proposed in the initial commentary on the DRT3 discovery. As of April 2026, all five remain solely conceptual: no construct based on DRT3 has been experimentally tested in any mammalian cell or disease model. None have been demonstrated empirically with the DRT3 system, and each is presented herein as a hypothetical application rather than a definitive product archetype. The natural DRT3 product is an alternating poly (GT/AC) DNA, which is unlikely to serve as the optimal pharmacological agent for any of these applications; engineering of the enzyme to generate alternative motifs would be necessary, and such engineering has not yet been documented. Figure 4 illustrates these application spaces alongside the engineering pipeline required to advance any of them to investigational study.

The application that has garnered the most attention concerns the local generation of immunostimulatory DNA within solid tumors, aimed at activating the cyclic GMP-AMP synthase (cGAS) and the stimulator of interferon genes (STING) pathway (Sun et al., 2013; Chen et al., 2016; Decout et al., 2021). Pharmacological STING agonists have entered oncology clinical trials with mixed outcomes; their short half-life, limited tumor penetration, and dose-limiting systemic inflammation have sparked interest in *in situ* strategies (Corrales and Gajewski, 2015; Kwon and Bakhoum, 2020; Lu et al., 2026). Enzymatic amplification and spatial confinement could, in principle, mitigate several of these limitations; however, two questions remain unresolved: whether the natural alternating poly(GT/AC) duplex activates cGAS, considering that cGAS recognition depends on both length and sequence context (Decout et al., 2021), and whether expression can be controlled in human cells. To date, no demonstration of such activation has been reported within a tumor model.

The remaining four proposed concepts, summarized along with their principal unresolved issues in Table 3, can be succinctly outlined. The application of antiviral agents would extend the phage-inhibitory paradigm to mammalian infections, where repetitive DNA sequences might compete for viral polymerase substrates or activate host nucleic acid sensors (Roers et al., 2016). The primary limitations are the lack of demonstrated efficacy against mammalian viruses and the potential for immunopathology resulting from broad innate immune activation. *In situ* transcription-factor decoys are designed to imitate factor-binding sites to sequester regulatory proteins such as nuclear factor kappa B (Mann and Dzau, 2000; De Stefano, 2011). Although this decoy approach has advanced to early clinical testing in a single indication (AMG0103, Phase 1b for chronic discogenic low back pain; Bruder et al., 2025), a DRT3-based variant would necessitate redesigning the Drt3b active site to encode a binding motif rather than poly(AC). *In situ* DNA biomaterials would leverage the self-association of locally concentrated repetitive DNA sequences into hydrogels or nanostructures (Seeman and Sleiman, 2018; Li et al., 2024; Ma et al., 2024), depending on achieving self-association thresholds intracellularly, on biodegradability, and on the creation of an unprecedented regulatory category. Vaccine co-formulation would involve co-delivering antigen mRNA with a DRT3 generator to generate a local adjuvant. However, since the natural product is not a CpG sequence, engagement of Toll-like receptor 9 would likely require an engineered CpG-producing variant (Krieg, 2002; Shirota et al., 2015; Jackson et al., 2018; Schillie et al., 2018). In all instances, the natural enzyme proves insufficient, and the critical challenge lies in active-site redesign, which has yet to be published.

### 6.3 Engineering requirements

The primary engineering requirement common to all applications is that the natural enzyme synthesizes only one motif. Any therapeutic application would necessitate either accepting this motif or modifying the enzyme to produce an alternative. There is no published evidence to suggest that the active site of Drt3b can be redesigned to generate arbitrary sequences; mutagenesis studies have identified conserved residues that enforce adenine-cytosine alternation, but a purposeful redesign to produce a specified alternative motif has not been documented (Deng et al., 2026). Therefore, programmability remains an open question rather than an existing capability. In addition to motif control, a practical platform would require regulated expression timing, cell-type-specific delivery, and self-limiting feedback mechanisms. These challenges are comparable to those addressed for CAR-T and mRNA-LNP platforms but have yet to be pursued in the context of DRT3.

### 6.4 Safety and regulatory considerations

A DRT3 product would deliver messenger RNA (mRNA) encoding a DNA-synthesizing enzyme, with the active species produced *in situ*. Its regulatory profile would resemble that of mRNA-LNP biologics but would necessitate an additional characterization package focused on the produced DNA, including length distribution, sequence purity, intracellular localization, persistence, and evidence against genomic integration. Existing Food and Drug Administration (FDA) frameworks for gene therapy and genome editing (Food Drug Administration, 2020, 2024a) provide only partial templates, as DRT3 neither edits the genome nor delivers a defined DNA species. Considerations such as biodistribution of the encoded enzyme to off-target tissues, genotoxicity through repair-mediated integration or aberrant recombination, off-target innate activation by both the mRNA and the DNA (with particular attention to type I interferonopathy phenotypes; Decout et al., 2021), and containment of expression in patients with predisposing inflammatory conditions must be thoroughly addressed. To date, none of these assessments has been conducted for DRT3. In certain respects, the regulatory package would exceed that of an mRNA vaccine, given that the active species is a polymer that requires comprehensive characterization.

Manufacturing would leverage lipid-nanoparticle and modified-mRNA processes validated at an industrial scale by COVID-19 vaccines (Karikó et al., 2008; Hou et al., 2021; Buschmann et al., 2021): capping, modified nucleotides (typically pseudouridine or N1-methylpseudouridine to limit innate sensing of the mRNA), polyadenylation, and a four-component ionizable-lipid formulation. The principal novelty would be the bicistronic or co-formulated delivery of two protein-coding mRNAs together with the non-coding RNA, with quality control to ensure that the complex assembles competently in target cells.

### 6.5 Comparative positioning against existing modalities

Table 2 situates the DRT3 platform within the broader context of modalities that exemplify the contemporary landscape of nucleic acid-based therapeutics. The platform occupies a distinctive intersection: it harnesses the delivery system characteristic of mRNA-LNP therapeutics, the active species category of oligonucleotide drugs, the protein expression platform employed in gene therapy, and the bacterial defense mechanism intrinsic to CRISPR-Cas systems. Nevertheless, it distinguishes itself from each of these modalities in several significant respects. Primarily, DRT3 remains in an initial preclinical conceptual stage rather than advancing through a formal development process; its programmability has yet to be empirically validated; and the active species is a polymer synthesized *in situ* rather than a predefined molecule administered.

**Table 2:** Comparative positioning of the DRT3 in situ platform against existing nucleic-acid-based therapeutic modal.

Modality	Active species and origin	Persistence	Sequence specificity	Stage and representative product
DRT3 *in situ* platform	Repetitive dsDNA generated intracellularly by mRNA-encoded enzyme	Bounded by mRNA decay and protein turnover	Motif-level only; programmability unproven	Preclinical concept; no *in vivo* demonstration (Deng et al., 2026)
CRISPR-Cas9 gene editing	Edited genomic DNA, by delivered ribonucleoprotein or mRNA	Permanent	Sequence-specific at gene level (programmable via guide RNA)	FDA-approved (exagamglogene autotemcel; Casgevy) for sickle cell disease (December 8, 2023) and transfusion-dependent beta-thalassemia (January 16, 2024)
Retron-based editing	Multicopy ssDNA donor for HDR, generated intracellularly	Editing event is permanent if integrated	Sequence-specific at gene level when paired with Cas effector	Preclinical research stage (Zhao et al., 2022; Lopez et al., 2022)
mRNA-LNP therapeutics	Translated protein from delivered mRNA	Bounded by mRNA decay	Determined by encoded protein	Approved (Comirnaty, Spikevax for COVID-19); investigational across multiple indications
Antisense oligonucleotides	Delivered chemically modified ssDNA or ssRNA	Days to weeks	Sequence-specific at transcript level	Multiple FDA approvals (e.g., nusinersen for SMA, eteplirsen for DMD)
siRNA/RNAi	Delivered short dsRNA or modified equivalent	Days to weeks per dose	Sequence-specific at transcript level	Approved (patisiran for hATTR amyloidosis; inclisiran for hypercholesterolemia)
CpG ODN immunostimulants	Delivered short ssDNA	Hours to days	Motif-level (CpG)	Approved (CpG-1018 as adjuvant in Heplisav-B); multiple investigational uses
STING agonists (small molecule/CDN)	Delivered small molecule or cyclic dinucleotide	Hours	Receptor-level binding	Investigational; multiple early-phase oncology trials with mixed results
Decoy ODN therapeutics	Delivered short dsDNA TF-binding mimic	Days to weeks	Motif-level (TF binding site)	Investigational (e.g., AMG0103 in chronic discogenic low back pain, Phase 1b complete; Bruder et al., 2025)

Stage and product information reflect the status of representative programs; readers are referred to current regulatory databases for the most up-to-date information. The DRT3 platform is at the preclinical-concept stage and has no in vivo demonstration as of this writing. The motif-level specificity entry for DRT3 refers to the natural enzyme, which produces only alternating poly (GT/AC); engineered variants that produce other motifs are hypothetical and have not been published. Programmability of the active site has not been established.

### 6.6 Hypothetical application spaces in summary

Table 3 systematically organizes the five speculative application domains and the corresponding engineering efforts required for each. The table serves as a concise summary rather than a compilation of specific programs. The rightmost column highlights the primary unresolved challenge for each domain; in each case, the challenge is significant, with the majority involving redesigning the Drt3b active site to generate a non-natural motif.

**Table 3:** Five speculative application spaces requiring prior proof of programmability and mammalian-cell function.

Speculative application space	Indication category	Mechanism (proposed)	Principal unsolved problem
Tumor innate-immunity engagement	Solid tumors resistant to checkpoint blockade	Local generation of cytosolic DNA, cGAS-STING activation, type I interferon induction	Whether natural product activates cGAS adequately, and whether expression can be contained spatially
Antiviral via host pathway engagement	Acute and chronic viral infections	Sequestration of viral factors and/or activation of host antiviral pathways	No demonstration of activity against any mammalian virus; risk of immunopathology
*In situ* transcription-factor decoys	Inflammatory and proliferative diseases	Intracellular generation of TF-binding-site mimic	Drt3b redesign to produce a TF-binding motif rather than poly(AC); not reported in any peer-reviewed or preprint literature
DNA biomaterials *in situ*	Localized depots, drug delivery, tissue support	Self-association of intracellularly generated repetitive DNA into structures	Concentration thresholds for self-association in human cells; biodegradation; novel regulatory category
Vaccine co-formulation	Pandemic and oncology vaccines	Co-delivered antigen mRNA plus DRT3 generator producing local DNA adjuvant	Drt3b redesign to produce CpG-containing motifs for TLR9 engagement; not reported in any peer-reviewed or preprint literature

Each row identifies an indication category, the proposed mechanism by which a DRT3-based platform might intervene, and the principal unsolved problem that distinguishes that space from a viable program. None of these applications has been demonstrated with the DRT3 system, and the natural enzyme product is unlikely to be the optimal pharmacological agent for any of them; engineering of the active site would be required, and that engineering is at present unpublished.

## 7 Open biological questions

Beyond the engineering challenges, numerous biological inquiries regarding the DRT3 system itself remain unresolved. The exact mechanism by which the synthesized poly (GT/AC) duplex facilitates phage defense has not been definitively established; while the proposed sponging and non-canonical-structure-recognition models are plausible, they lack empirical validation (Deng et al., 2026). The function of ST61 as a trigger and the question of whether DRT3 constitutes a class of immune systems that detect specific viral signatures require further investigation. Additionally, the extent of DRT3 distribution across bacterial taxa and whether all DRT3 systems share a common architecture or exhibit subtypes with varying motif outputs remains to be thoroughly characterized. The relationship between DRT3 and the numerous other UG-class reverse transcriptases identified in the broader bacterial defense survey remains not fully elucidated (Mestre et al., 2022). Each of these issues bears significant implications for translational applications, as a more comprehensive understanding of the natural system will guide the strategic engineering decisions.

A secondary issue pertains to the mechanism of protein-directed nucleotide selection itself. The publication by Deng et al. (2026) identifies the conserved residues responsible for enforcing nucleotide alternation but does not yet offer a quantitative model that explains how the active-site geometry distinguishes between adenine and cytosine relative to other bases. A detailed, residue-by-residue understanding of selectivity will be necessary prior to attempting rational redesign. Additionally, a comparative structural analysis of Drt3b across various bacterial lineages, specifically seeking natural variants with differing active site configurations at pertinent positions, is likely to constitute a productive subsequent step. The question of whether the principle of protein-directed nucleotide selection is exclusive to DRT3 or extends to other DRT subtypes and enzymes outside this family remains for future investigation.

## 8 Discussion and outlook

The discovery of DRT3 establishes a previously undocumented mechanism of biochemical specificity in DNA synthesis: protein-directed nucleotide selection, wherein the geometry of a protein active site enforces sequence specificity without the need for an opposing nucleic acid template. More cautiously stated, yet equally significant, it broadens the biochemical possibilities of sequence-specific DNA synthesis, without constituting protein-to-nucleic-acid information transfer in the sense proposed by Crick, whilst preserving the fundamental base-pairing logic inherent in conventional polymerases. The natural product is a fixed alternating poly (GT/AC) duplex; the system identified is narrowly biochemically. However, the implications are extensive, as the demonstration that protein architecture can encode the sequence of a DNA product broadens the theoretical framework of synthetic biology and proposes engineering strategies that had hitherto been unprecedented (Deng et al., 2026).

Three primary avenues of research are expected to be of most significance. The first involves the structural and biochemical extension of the DRT3 mechanism to other DRT subtypes, for which the DNA products and mechanisms of action remain unidentified (Mestre et al., 2022; Wang et al., 2025; Figiel et al., 2026 (preprint). It is plausible that additional variants of protein-directed selection exist within the DRT family, and a systematic mechanistic survey is likely to uncover them. The second avenue concerns engineering the Drt3b protein to modify its sequence specificity, which is critical for nearly all therapeutic applications discussed in this review. This research has not yet been published. The third avenue involves preclinical characterization of *in situ* DNA-generation strategies in disease models, with initial demonstrations most likely conducted in murine tumor or viral infection models. Until such preclinical data are available, the discussion of therapeutic applications shall remain conceptual.

It is advisable to conclude with a note of epistemic humility. The history of nucleic-acid-based therapeutics demonstrates that many platforms have initially generated significant enthusiasm within the field but required 15 to 20 years to be transformed into tangible products. CRISPR-Cas9 was first characterized in its modern, programmable form in Jinek et al. (2012), and the first FDA-approved gene-editing therapy utilizing this technology, exagamglogene autotemcel (Casgevy), received FDA approval for sickle cell disease on December 8, 2023, and for transfusion-dependent beta-thalassemia on January 16, 2024 ((Food Drug Administration, 2023, 2024b)). This spans 11 years, despite the platform being among the most extensively funded endeavors in contemporary biotechnology development. Retrons, originally identified in the 1980s, have only recently been adapted as gene-editing tools for human cells within the past 5 years (Inouye et al., 1989; Zhao et al., 2022; Lopez et al., 2022). The development of mRNA therapeutics spanned multiple decades, including a crucial breakthrough involving modified nucleotides that enabled the technology to function without triggering innate immune responses (Karikó et al., 2008), with deployment accelerating following the COVID-19 pandemic. Given this context, the pragmatic outlook for the DRT3 platform suggests that proof-of-concept therapeutic products could emerge within the next 5 to 10 years for the most tractable indications, with broader application likely requiring significantly longer periods. The platform is established, the scientific foundation remains robust, and the conceptual landscape is expansive; however, engineering efforts are still in their early stages.

The profound significance of the DRT3 discovery may lie less in its specific therapeutic applications and more in its ability to broaden the theoretical framework of synthetic biology. The understanding that protein structures can function as templates for DNA synthesis provides a valuable design insight, implying that alternative templating methods, including those involving mineral surfaces, self-assembled peptide nanostructures, or engineered ribonucleoprotein scaffolds, may be attainable through biochemical means. If the DRT3 mechanism is regarded as the inaugural instance of a broader category, its long-term influence will likely extend to changes in the pedagogy of molecular biology and to the development of pharmaceuticals. Presently, this system serves as a reminder that, despite 70 years of rigorous study, the fundamental chemistry underlying DNA synthesis continues to reveal unforeseen complexities (Watson and Crick, 1953; Deng et al., 2026).
